# A Large Retrospective Study of 12714 Cases of LEEP Conization Focusing on Cervical Cancer That Colposcopy-Directed Biopsy Failed to Detect

**DOI:** 10.1155/2018/5138232

**Published:** 2018-04-30

**Authors:** Qing Cong, Yu Song, Qing Wang, Hongwei Zhang, Shujun Gao, Ming Du, Feng Xie, Jing Dong, Hua Feng, Wenjing Diao, Caiying Zhu, Long Sui

**Affiliations:** ^1^Obstetrics and Gynecology Hospital of Fudan University, Shanghai, China; ^2^Shanghai Key Laboratory of Female Reproductive Endocrine Related Diseases, Shanghai, China; ^3^Shanghai Medical Center of Key Programs for Female Reproductive Diseases, Shanghai, China

## Abstract

Punch biopsy is important in the diagnosis of cervical cancer. However, it may fail to detect early cervical cancers. A retrospective study was performed in the largest academic women's hospital in China to demonstrate cervical cancer that colposcopy-directed biopsy failed to detect.* Methods*. Patients who were diagnosed with high-grade squamous intraepithelial lesion (HSIL), adenocarcinoma in situ (AIS), and persistent low-grade squamous intraepithelial lesion (LSIL) via colposcopy-directed biopsy and had further undergone loop electrosurgical excision procedure (LEEP) conization were included. These procedures were performed at Obstetrics and Gynecology Hospital of Fudan University from July 1, 2013, to December 31, 2016. In total, 5.98% (760/12714) of patients who underwent conization were diagnosed with invasive cervical cancer. Persistent LSIL (0.24%), HSIL (6.37%), and AIS (24.31%) were detected cancer by conization. Histological subtypes included squamous cell carcinoma (92.0%), adenocarcinoma (5.1%), adenosquamous carcinoma (1.8%), adenoid basal type carcinoma (0.9%), and small cell neuroendocrine carcinoma (0.1%). Cytology reports consisted of HSIL (45.4%), atypical squamous cells of undetermined significance (ASC-US) (16.1%), and LSIL (11.6%), and atypical squamous cells cannot exclude HSIL (ASC-H) (9.3%), squamous cell carcinoma (0.9%), AGC (atypical glandular cells, 0.9%), AIS (0.4%), and NILM (negative for intraepithelial lesion or malignancy, 15.4%). The sensitivity of high-risk human papillomavirus (hrHPV) screening (96.4%) was significantly higher than that of cytology (84.6%) (*P* < 0.01), with sensitivity of cotesting at 99.8% and a ratio of double-negative results at 0.2%. The sensitivity of cytology and hrHPV screening of different cervical cancer histologic subtypes was also demonstrated. In this large retrospective study, we systematically reported the cytology, hrHPV, pathology, and stages of cervical cancer that colposcopy-directed biopsy failed to detect.

## 1. Introduction

Cervical cytology and high-risk human papillomavirus (hrHPV) screening greatly contribute to the early detection of cervical cancer and precancers such as high-grade squamous intraepithelial lesion (HSIL) or cervical intraepithelial neoplasia 2/3 (CIN2/3) [1]. Colposcopy has played a pivotal role in reducing the incidence and mortality from cervical cancer over the past 50 years [2, 3]. In CIN2/3 detected by punch biopsy, LEEP conization allows further and more accurate histologic examination of the transformation zone [4]. Although it goes undetected by visual inspection of the naked eye or colposcopy-directed biopsy, unsuspected invasive cancer can be detected by histopathologic examination of conization masses. In conization, loop electrosurgical excision procedure (LEEP) conization, also known as large loop excision of the transformation zone (LLETZ), high-frequency-needles, and laser conization are equally optimal, whereas cold-knife conization is associated with an excessive risk for subsequent obstetric complications [5].

Cervical precancers can be treated or even examined for invasive cancers through conization. Treatment management of invasive cervical cancer and its various stages completely differs from that of precancer. Given the wide range of treatment recommendations, accurate diagnosis of cervical precancer and cancer is essential and cervical conization should be given preference over hysterectomy in cases of precancer [6]. Studies have reported that 2.50% (1/40)–17.39% (8/46) of CIN3 punch biopsies and none (0/94–0/24) of CIN2 punch biopsies had invasive cancer [4, 7–9]. The ratio of unsuspected, invasive cervical cancer cases to precancerous lesions within these studies differs greatly. Until now, there is no systematic study of these cancers. To gain a deep understanding of these early cervical cancers that failed to be diagnosed by colposcopy-directed biopsy, we retrospectively analyzed 12714 cases of consecutive LEEP conization in the largest Obstetrics and Gynecology Hospital in China.

## 2. Materials and Methods

### 2.1. Patients

Patients who underwent cervical LEEP conization in Obstetrics and Gynecology Hospital of Fudan University (OGHFU) were included from July 1, 2012, to December 31, 2016. In OGHFU, patients with abnormal cervical cytology or positive hrHPV testing were referred to colposcopy in 2–6 weeks. Colposcopy-directed biopsy was performed on all patients by experienced colposcopists. HSIL, adenocarcinoma in situ (AIS), and low-grade squamous intraepithelial lesion (LSIL) (persistent for 2 or more years or LSIL with cytology of HSIL/atypical squamous cells cannot exclude HSIL [ASC-H]/atypical glandular cells [AGC]/AIS) diagnosed by punch biopsy were subjected to LEEP conization. In addition, one patient with heavy watery vaginal discharge who was diagnosed with cervicitis via punch biopsy also underwent LEEP conization.

### 2.2. Cytology and hrHPV Testing

In cytology testing, liquid based cytology (ThinPrep [Hologic, Massachusetts, USA] and SurePath [Becton, Dickinson and Company, New Jersey, USA]) were used. In hrHPV testing, the Hybrid Capture 2 (HC2) method (Qiagen, Limburg, Netherlands) was used for the detection of high-risk and intermediate-risk HPV types 16, 18, 31, 33, 35, 39, 45, 51, 52, 56, 58, 59, and 68.

### 2.3. LEEP Conization and Pathologic Examination

All the procedures were performed by one of 18 staff colposcopists. Different diathermy loops were used depending on the size of cervical lesions to excise and location of the transformation zone. All excisions were performed under colposcopic guidance. The cervical transformation zone and lesion excised to an adequate scale, extending 4 to 5 mm beyond the lesion in most cases. The tissues were removed to a depth of 7–10 mm, 10–15 mm, and 15–25 mm in type I, II, and III cervical transformation zone, respectively. A second pass with a small loop can also be performed to obtain an endocervical specimen for further histologic evaluation. Information on loop size, volume, length, and thickness of the cone specimen was recorded. For each cone, the pathologists cut the cone tissue into 12 pieces and embedded each piece into a paraffin block. Both ectocervical and endocervical margins were clearly read and reported by pathologists. All pathologic specimens were processed by a standardized protocol, interpreted by an experienced staff pathologist and then verified by another advanced pathologist.

### 2.4. Statistical Analysis

Approval was obtained from the Institutional Review Board of OGHFU before the data extraction was performed, and all patients gave consent to research. The Pearson chi-square test was used for statistical analysis and conducted using SPSS 16.0 (SPSS Inc., Chicago, Illinois, USA). A *P* value < 0.05 was considered statistically significant.

## 3. Results

In total, 12714 consecutive patients of HSIL, AIS, and LSIL diagnosed by colposcopy-directed biopsy underwent LEEP conization. As a result, 5.98% (760/12714) were further diagnosed with invasive cervical cancer.

In Table 1, the pathology of 759 patients of cervical cancer before and after LEEP conization was shown, excluding one patient who was diagnosed with cervicitis via punch biopsy. By LEEP conization, 0.24% of LSIL, 6.37% of HSIL and 24.31% of AIS diagnosed by punch biopsy were further confirmed as having cervical cancer. Cervical cancer was detected via LEEP cone biopsy in 35 of 144 (24.31%) patients with AIS in biopsy. Of these 35 patients, 82.9% (29/35) had adenocarcinoma, 14.3% (5/35) had adenosquamous carcinoma, and 2.8% (1/35) had small cell neuroendocrine carcinoma. The ratio of cervical cancer in LSIL was significantly lower than HSIL (*P* < 0.01). The ratio of cervical cancer in AIS was significantly higher than HSIL (*P* < 0.01).

From Table 2, the mean age of 760 patients was 44 ± 9 years (range: 22–70). Five histological subtypes of cervical cancer were detected, including squamous cell carcinoma (92.0%), adenocarcinoma (5.1%), adenosquamous carcinoma (1.8%), adenoid basal type carcinoma (0.9%), and small cell neuroendocrine carcinoma (0.1%). The mean ratios of stages IA1, IA2, and IB1 were 58.6%, 2.4%, and 39.1%, respectively.

Available cytology and hrHPV tests of cervical cancer were shown in Table 3. Diverse cytology was seen in reports of cervical cancer, including HSIL (45.4%), ASC-US (16.1%), LSIL (11.6%), ASC-H (9.3%), squamous cell carcinoma (SCC, 0.9%), AGC (0.9%), AIS (0.4%), and NILM (negative for intraepithelial lesion or malignancy, 15.4%). In 259 cases of HSIL, 17 were HSIL (with possibility of cancer) and 2 were HSIL admixed with AGC. In 88 cases of NILM, 82 had available cotesting of hrHPV. These were 98.8% hrHPV positive and 1.2% hrHPV negative. The sensitivity of hrHPV was significantly higher than cytology (96.4% versus 84.6%, *P* < 0.01). In 19 cases of negative hrHPV, 18 available cytology reports were 5 HSIL, 4 ASC-US, 3 LSIL, 2 ASC-H, 2 AGC, 1 SCC, and 1 NILM. One of 760 (0.13%) patients had double-negative results of both cytology and hrHPV.

Table 4 showed that 463 cervical cancer cases diagnosed by LEEP conization had both cytology and hrHPV results. Among them, 78.6% were both cytology and hrHPV positive, 17.5% were only hrHPV positive, 3.7% were only cytology positive, and 0.2% were double negative. The sensitivity of cotesting was 99.8%.

In Table 5, the sensitivity of cytology and hrHPV screening for cervical cancer in different histology subtypes was shown. The sensitivity of cytology screening was 85.8% for squamous cell carcinoma, 65.6% for adenocarcinoma, 75.0% for adenosquamous carcinoma, and 80% for adenoid basal cell carcinoma. The sensitivity of hrHPV screening was 96.9% for squamous cell carcinoma, 91.2% for adenocarcinoma, 88.9% for adenosquamous carcinoma, 100% for adenoid basal cell carcinoma, and 100.0% for neuroendocrine carcinoma.

## 4. Discussion

Until now, there were few studies examining how frequently cervical cancer was detected in AIS and HSIL cases diagnosed by punch biopsies. A few earlier studies indicated that 2.50% (1/40), 2.63% (1/38), 3.70% (2/54), and 17.39% (8/46) of CIN3 cases were found via conization to have an underlying, unsuspected invasive cancer, respectively [4, 7–9]. Xiang et al. reported 6.74% (77/1142) of HSIL punch biopsies were diagnosed with cancer by conization [10]. Our study reported cervical cancer in 24.31% (35/144) of punch biopsies detecting AIS and 6.37% (721/11313) of those detecting HSIL, which is the largest retrospective study to date.

In summary, the ratio of cervical cancer confirmed by LEEP conization in AIS was significantly higher than HSIL. In comparison with sufficient inspection of squamous epithelia, which were located on the surface of cervix, most glandular epithelia were in the cervical canal and stroma where crypts were formed. Hence, all glandular epithelia cannot be thoroughly inspected in colposcopy. In addition, punch biopsy and even endocervical curettage could fail to supply enough glandular samples because the early lesions might be in the crypts. Therefore, the difference of histology could be the main reason why a higher rate of adenocarcinoma was detected by excisional procedure.

The ratio of cervical cancer diagnosed by LEEP conization in punch biopsies of LSIL was extremely low (0.24%, 3/1257). Among these cases, 1 was diagnosed as microinvasive squamous cell carcinoma with unavailable cytology while the other 2 were diagnosed as invasive squamous cell carcinoma (IB1) with HSIL cytology. This demonstrates that cancer diagnosis cannot be excluded from HSIL cytology readings and HSILs should be treated by excisional procedures. In fact, a study performed by Kietpeerakool et al. found occult invasive lesions of the cervix in 17% of women with HSIL Pap smears who underwent a “see and treat” approach [11].

In cervical cancers that punch biopsy failed to detect, squamous cell carcinoma remained the common subtype, followed by adenocarcinoma, adenosquamous cell carcinoma, adenoid basal cell carcinoma, and neuroendocrine carcinoma. Notably, the mean age of adenoid basal cell carcinoma was 63 years, which was significantly greater than the rest of the ages ranging from 37 to 44 years (*P* < 0.01). The ratio of IA1 cases to total cases of the same pathology, in descending order, was squamous cell carcinoma (62.7%), adenoid basal cell (14.3%), adenocarcinoma (12.8%), adenosquamous (7.1%), and neuroendocrine (0%). Hence, squamous cell carcinoma is the most common cervical cancer with the majority being IA1.

There were considerable differences among global laboratories in the sensitivity of cytology screening. In the ATHENA (Addressing the Need for Advanced HPV Diagnostics) study, the sensitivity of cytology varied from 42.0 to 73.0% in CIN grade 2 or worse (CIN2+) [12]. In the cervical cancer screening results among 256,648 women in multiple clinical practices, the sensitivity of cytology was 90.9% in CIN2/3 and 93.1% in CIN3 [13]. The sensitivity of hrHPV was 95.8% in CIN2/3 and 98.8% in CIN3 [13]. Thus, hrHPV is more sensitive than cytology in precancer screening (*P* < 0.01 in both CIN2/3 and CIN3). In early cervical cancers that punch biopsy failed to detect, our study showed the sensitivity of cytology and hrHPV was 84.6% and 96.4%, respectively, which indicated the sensitivity of hrHPV was significantly higher than that of cytology in screening early cervical cancer. In all invasive cervical cancers, the sensitivity of cytology screening (84.5%–89.9%) [13–15] is similar to the sensitivity of hrHPV screening (81.4%–92.5%) [14–16]. This demonstrated that the sensitivity of hrHPV decreased as cancer progressed, probably because of the difficulty in detecting hrHPV in necrotic and bleeding tumor samples. Compared with early cervical cancer, the sensitivity of hrHPV screening for all invasive cancers was significantly lower (92.5% versus 96.4%, *P* = 0.01) while the sensitivity of cytology screening was constant (84.5% versus 84.6%, *P* = 0.98). This suggests that hrHPV screening could help detect more early cervical cancer compared to cytology. In addition, the sensitivities of cytology and hrHPV in different histology subtypes are different from each other. Adenocarcinoma and adenosquamous carcinoma had relatively lower sensitivity of cytology and hrHPV screening compared to other subtypes.

**Figure 1:**
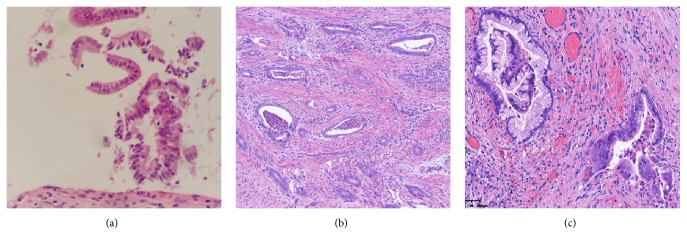
The patient complained of heavy, watery vaginal discharge with negative cytology and hrHPV results. Pathological examination of the punch biopsy and endocervical curettage showed cervicitis (a). Then, histologic examination of the LEEP cone biopsy showed minimal deviation adenocarcinoma (MDA) (b, c).

In our study, 0.13% (1/760) of patients had negative cytology and hrHPV results. This patient complained of heavy, watery vaginal discharge. Punch biopsy and endocervical curettage showed cervicitis (Figure 1(a)). Since watery vaginal discharge continued to increase, LEEP cone biopsy was performed and the patient was diagnosed with minimal deviation adenocarcinoma (MDA) (Figures 1(b) and 1(c)). Pathologists reviewed punch biopsy and endocervical curettage again and corrected the diagnosis to atypical glands. MDA is rare, consisting of 1–3% of all cervical adenocarcinomas. MDA is an endocervical adenocarcinoma, which is mucinous and well differentiated. It consists of an endocervical glandular hyperplasia of lobular architecture that resemble glands, but with the characteristics of adenocarcinoma [17]. MDA is associated with the autosomal dominant disease Peutz-Jeghers syndrome (PJS). PJS is characterized by the development of benign hamartomatous polyps in the gastrointestinal tract and hyperpigmented macules on the lips and oral mucosa with mutations in the STK11 gene [18]. MDA is usually hrHPV negative and undetectable by punch biopsy [19]. Hence, in cases of persistent or increasing heavy watery vaginal discharge, cervical conization should be performed to exclude cervical adenocarcinoma, even if cotesting and punch biopsy are normal. Furthermore, in all cervical cancers, Tao et al. reported 3.9% (9/231) of patients have double-negative results. Compared with squamous cell carcinoma, adenocarcinoma has significantly higher rates of prior negative results with both hrHPV and Pap cytology [14].

## Figures and Tables

**Table 1:** Pathology of cervical cancer before and after LEEP conization.

Punch biopsy	Conization				Number of LEEP conizations	% of cervical cancers
	IA1	IA2	IB1	Total		
LSIL	1	0	2	3	1257	0.24%
HSIL	439	16	266	721	11312	6.37%
AIS	5	2	28	35	144	24.31%
Total	445	18	296	759	12713	5.97%

*Note*. The difference between any 2 groups was statistically significant (*P* < 0.01).

**Table 2:** Histological subtypes, ages, and stages of cervical cancer diagnosed by LEEP conization.

Histological subtypes	Mean age (range)	IA1 *n* (*n*/total^h^)	IA2 *n* (*n*/total^h^)	IB1 *n* (*n*/total^h^)	Total^h^ (*n*/total)
Squamous cell ca	44 (22–70)	438 (62.7%)	17 (2.4%)	244 (34.9%)	699 (92.0%)
Adenocarcinoma	42 (29–59)	5 (12.8%)	1 (2.6%)	33 (84.6%)	39 (5.1%)
Adenosquamous ca	42 (32–57)	1 (7.1%)	0 (0%)	13 (92.9%)	14 (1.8%)
Adenoid basal ca^a^	63 (56–68)	1 (14.3%)	0 (0%)	6 (85.7%)	7 (0.9%)
Neuroendocrine ca	37 (37-37)	0 (0%)	0 (0%)	1 (100%)	1 (0.1%)
Total	44 (22–70)	445 (58.6%)	18 (2.4%)	297 (39.1%)	760 (100%)

^a^Four of 7 (57.1%) adenoid basal carcinomas admixed with squamous cell carcinoma. ^h^Total number of IA1, IA2, and IB1 of the same histological subtype.

**Table 3:** Cytology and hrHPV tests of cervical cancer diagnosed by LEEP conization.

Tests	Number of cases	%
Cytology	570	100.0%
HSIL	259	45.4%
ASC-US	92	16.1%
LSIL	66	11.6%
ASC-H	53	9.3%
SCC	5	0.9%
AGC	5	0.9%
AIS	2	0.4%
NILM	88	15.4%
hrHPV	534	100.0%
Positive	515	96.4%
Negative	19	3.6%

**Table 4:** Cotesting results of cervical cancer diagnosed by LEEP conization.

Category	hrHPV positive	hrHPV negative	Total
Cytology positive	364 (78.6%)	17 (3.7%)	381 (82.3%)
Cytology negative	81 (17.5%)	1 (0.2%)	82 (17.7%)
Total	445 (96.1%)	18 (3.9%)	463 (100.0%)

**Table 5:** The sensitivity of cytology and hrHPV screening of cervical cancer in different histological subtypes diagnosed by LEEP conization.

Histological subtypes	Cytology			hrHPV		
	Number of abnormal cases	Number of tests	Sensitivity	Number of positive cases	Number of tests	Sensitivity
Squamous cell ca	447	521	85.8%	469	484	96.9%
Adenocarcinoma	21	32	65.6%	31	34	91.2%
Adenosquamous ca	9	12	75.0%	8	9	88.9%
Adenoid basal cell ca	4	5	80.0%	6	6	100.0%
Neuroendocrine ca	NA	1	NA	1	1	100.0%
Total	481	570	84.4%	515	534	96.4%
